# Browse or browsing: Investigating goat preferences for feeding posture, feeding height and feed type

**DOI:** 10.3389/fvets.2022.1032631

**Published:** 2022-11-30

**Authors:** Marjorie Cellier, Birte L. Nielsen, Christine Duvaux-Ponter, Hannah B. R. Freeman, Rina Hannaford, Briar Murphy, Emma O'Connor, Kevan R. L. Cote, Heather W. Neave, Gosia Zobel

**Affiliations:** ^1^Université Paris-Saclay, INRAE, AgroParisTech, UMR Modélisation Systémique Appliquée aux Ruminants, Paris, France; ^2^Universities Federation for Animal Welfare (UFAW), Wheathampstead, St. Albans, United Kingdom; ^3^Animal Behaviour and Welfare Team, AgResearch Ltd., Ruakura Research Centre, Hamilton, New Zealand; ^4^Data Science Team, AgResearch Ltd., Grasslands Research Centre, Palmerston North, New Zealand; ^5^Department of Animal Science and Veterinary, Aarhus University, Aarhus, Tjele, Denmark

**Keywords:** feeding behavior, dairy, natural behavior, welfare, grass, leaves

## Abstract

Goats naturally browse different forages in various postures; this differs from typical farm practice, thus there are opportunities to improve goat welfare by understanding what and how they like to eat. We investigated if feeding preference was related to posture, feeder height relative to the ground, and type of feed. Sixteen adult, Saanen cross females participated in two experiments comparing a floor-level feeder (grazing posture; farm standard), with an elevated feeder (browsing posture; Exp1) and a platform-level feeder (raised, grazing posture; Exp2), when two forages (leaves, grass) were offered. Measurements included feed intake (g of DM/feeder), feeder switching frequency, first feeder visited, latency to visit first feeder and exploration and non-feeding activity time. Effects of posture (Exp1), height (Exp2) and feed type were analyzed. Type of feed affected preference for feeding posture and height. All goats consumed leaves over grass (Exp1: POP: 188 ± 6.52 g, GRA: 20.3 ± 7.19 g; Exp2: POP: 191 ± 6.15 g, GRA: 0.231 ± 6.91 g; *P* < 0.001), and the feeder containing leaves was often visited first (Exp 1: GRA/POP: 94% of visits, *P* < 0.001, POP/GRA: 53%, *P* = *0.724*; Exp 2: GRA/POP: 91%, *P* < 0.001; POP/GRA: 69%, *P* = 0.041). When goats received only leaves, they consumed more from the floor-level (162 ± 22.2 g) vs. elevated level (102 ± 21.9 g) feeder (*P* = 0.039). When goats received only grass, there was no posture or height preference; however, they changed feeders more frequently (at least 4x (Exp1) and 2x (Exp2) more than other combinations; *P* > 0.01). Feed intake was negatively affected by exploring time (Exp1 only: r = −0.541; *P* < 0.001) and performing non-feeding activities (Exp1: r = −0.698; *P* < 0.001; Exp2: r = −0.673; *P* < 0.001). We did not identify a preference for elevated feeding posture; however, we suggest that our short test (compared to previous work) encouraged goats to make choices based on line-of-sight and also that the elevated feeder design (replicated from previous work) made leaf access harder. Nonetheless, we highlight that some goats actively used the elevated feeder; this coupled with the clear preference for leaves over grass, suggests that offering feed type and presentation diversity would allow individuals to express their natural feeding behavior more fully.

## Introduction

Society is becoming increasingly interested in the welfare of farmed animals with an growing appreciation of animal welfare parameters over other quality attributes of food products (1). Indoor housing of ruminants is often criticized because of perceived intensiveness and lack of naturalness. Providing animals with a “good life” (2) requires more than just ensuring animals are healthy and productive. In this context, the French Agency for Food, Environmental and Occupational Health & Safety (3) developed the following definition of animal welfare: “*The welfare of an animal is the positive mental and physical state related to the satisfaction of its physiological and behavioral needs as well as to its expectations. This state varies according to the animal's perception of the situation*.” One of the means for satisfying the behavioral needs of animals is to consider their natural behavior. Bracke and Hopster (4) defined natural behavior as the behavior animals tend to adopt when given the opportunity under natural conditions. For an animal to function as they evolved to do in their natural environment, they should be able to perform natural behaviors. Depending on the animal, these may include foraging, exploration, play and grooming (4). These are important for animal welfare because natural behaviors satisfy needs for which the animal is motivated and are likely to involve patterns which are associated with positive states (5). Commercial dairy goat farming systems, more precisely indoor housing systems, have focused on promoting good production and health outcomes, and thus often fail to provide opportunities for naturalness. We suggest that housing alterations are possible, as well as feasible, particularly with regard to feeding management. To make such changes, it is important to understand which opportunities goats value most in their environment.

Under natural conditions, goats show great adaptation and flexibility in their feeding behavior. For example, Shi et al. (6) showed that feeding duration in feral goats was influenced by both the time of year (with the lowest level in late summer) and the time of day (crepuscular, with an additional less obvious midday peak). Preference for certain plant species by feral goats was also different across seasons (7). Goats are classified as both browsers and grazers (8), and actively forage at different heights. Goats have also been observed feeding while perched in trees (9). This inclusion of the third dimension (height) when foraging has advantages, as it widens the foraging zone, and eating at eye-level may reduce parasite and predator risks (10, 11) by allowing goats to be more alert and to better monitor the environment. Sanon et al. (12) also reported that goats could browse up to a height of 2.1 m, more than twice the height of their bodies.

Opportunities for goats to express their natural feeding behavior, such as adopting browsing posture and feeding at height, in a conventional farm system are limited. In commercial systems, the feed is usually provided on the ground outside a feed rail, resulting in a posture that resembles that of grazing, and the design of the feed rail varies depending on whether the goats are horned or not (13, 14). However, these systems may increase discomfort and risk of injury related to the feeding barrier; these injuries can include skin lesions and stress on joints and hooves for example, and thus may negatively affect animal welfare (15). Although if it is poorly designed an elevated feed bunk could also create risk of injury. Nonetheless, elevation of the feed may be an important contributor to promote natural browsing behavior. Indeed, goats naturally prefer to eat feed presented 20 to 120 cm above the ground (16). Van et al. (17) suggested that hanging foliage was the best way to improve consumption in goats. Later, Neave et al. (18) demonstrated that young Saanen cross female goats consumed more feed when given the opportunity to eat from an elevated feeder that promoted a browsing body posture, compared to a floor feeder mimicking the grazing posture. Feeding posture may not be the only factor influencing the feeding behavior of goats given their preference to position themselves at elevated height (19), so the height above ground whilst feeding may also have an influence. Indeed, Aschwanden et al. (20) found that allowing goats to eat whilst standing on a raised platform increased feeding time compared to feeding from the floor.

Adding to the complexity of the feeding system is not just how the feed is delivered, but also the type of feed that is provided. To achieve high levels of milk production, modern diets are typically rich in concentrates and are predominantly homogenous [e.g., (21, 22)]; these diets rarely contain any form of browse (i.e., shoots, twigs, leaves of trees and shrubs), thus limiting the possibilities of feed selection by goats (23).

In order to better understand which environmental changes related to feeding management might be most beneficial for promoting the natural behavior of goats and thus better welfare, this study aimed to investigate preference for feeding posture and feeding height and to determine to what extent the preference was dependent upon feed type. To examine feeding posture and feeding height preferences, we conducted two experiments. In experiment 1, we compared two feeding postures: a control floor-level feeder allowing a grazing posture, and an elevated-level feeder allowing a browsing posture. In experiment 2, we compared two feeding heights while maintaining the same feeding posture: the control floor-level feeder and the same feeder but raised off the ground (platform-level feeder); both of these feeders encouraged a grazing posture. In both experiments, two types of forage (leaves or grass) were offered in the feeders.

We hypothesized that preference would be in the following order (most preferred to least preferred): leaves consumed in browsing posture, leaves consumed in grazing posture, grass consumed in browsing posture, and grass consumed in grazing posture (Exp1). Similarly, we hypothesized that goats would preferentially use the raised option when only grazing posture was available (Exp2). We also hypothesized that the duration of exploratory behavior would differ depending on the combinations of feeding height/posture and feed type offered. More precisely, the goats would explore more when grass was available in order to search for another source of feed, without this searching behavior being related to another feeding height/posture.

## Materials and methods

The study was conducted from February to March 2019 at the Ruakura Research Centre in Hamilton, New Zealand. All procedures were approved by the Ruakura Animal Ethics Committee (Hamilton, New Zealand: no. 14680) under the New Zealand Animal Welfare Act 1999.

### Animals, housing, and diet

Sixteen female (non-lactating, non-pregnant), disbudded Saanen cross goats (four to six years old; mean (± SD) body weight: 75.4 ± 10.6 kg) were enrolled from a herd of 26 animals. Prior to the experiment, the goats were on pasture 24 h/day, with access to a shelter. Eight days prior to the beginning of testing, goats were habituated to the experimental housing and feeding schedule.

At 0900 h on habituation and testing days, the goats were moved into four adjacent pens (each approximately 8 m^2^), bedded with wood shavings, equipped with a hay rack and with *ad libitum* access to water. Goats were randomly assigned to testing order and were always kept in the same groups of four in order of test appearance (i.e., goats 1–4 in pen 1, goats 5–8 in pen 2, etc.). Testing took place between 1000 and 1600 h each day. Goats were tested in two batches: half of the goats in the morning and the other half in the afternoon, maintaining the order throughout the study. Hay was provided in the pens in sufficient amounts to ensure continuous access during the time spent there, thus reducing the risk that testing was impacted by hunger. Upon completion of testing, goats were returned to pasture with the rest of the experimental herd for the rest of the day. Minimal pasture availability due to dry summer conditions resulted in the herd being contained overnight (beginning at 1630 h minimum) in an area of approximately 148 m^2^ with a covered shelter of 52 m^2^ and provided with approximately 0.7 kg/goat of meadow hay (comprised of ryegrass and white clover) in a bale-sized hay rack.

### Experimental design and test procedure

**Figure 1:**
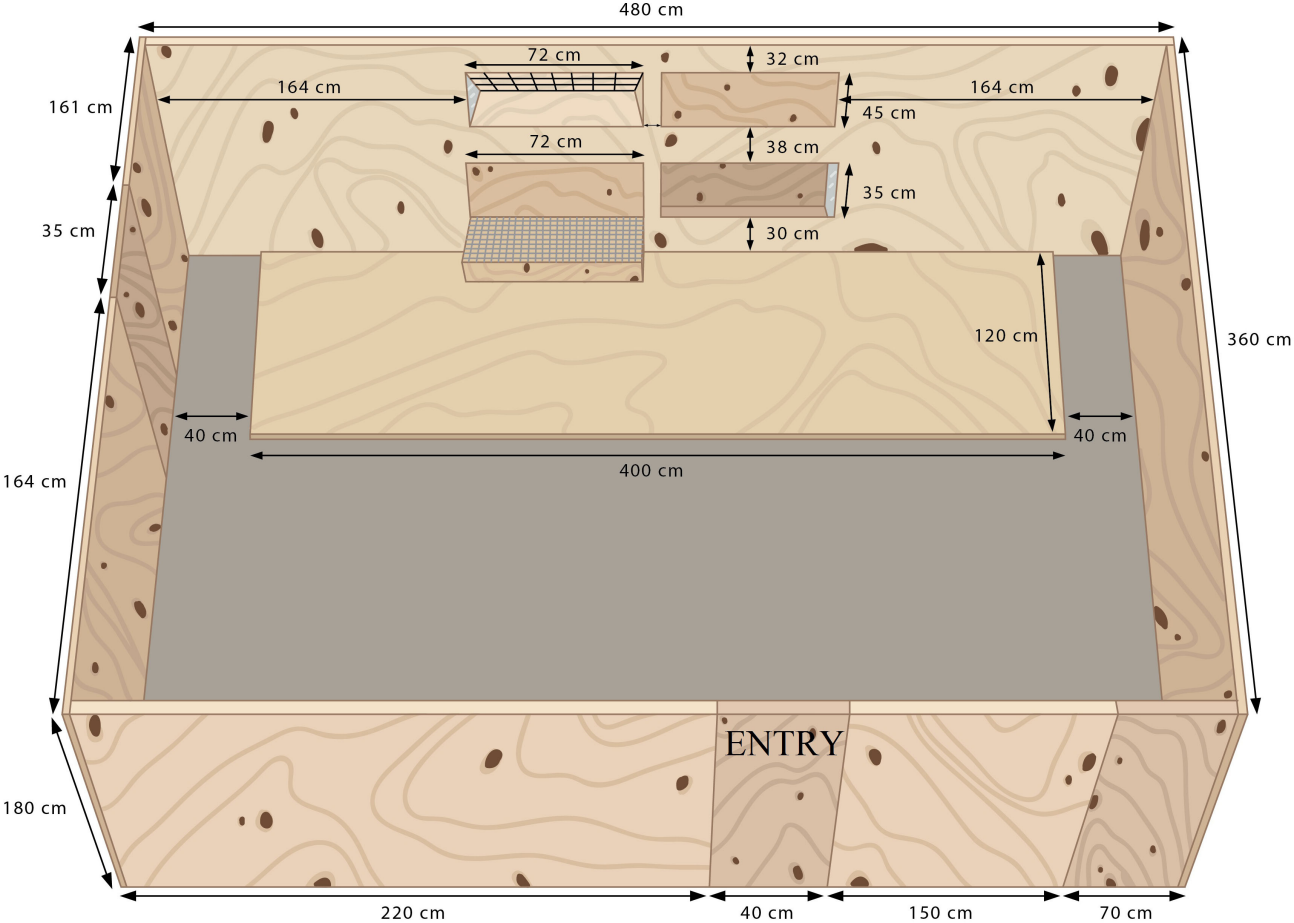
Layout of the testing arena with solid plywood walls and a concrete floor that was partially covered by plywood directly in front of the feeders. It contained feeders which could be opened or closed (elevated feeders accessible by a movable step, floor-level feeders). All feeders were made of plywood boxes with a plate of acrylic glass at the end, but in the elevated feeder the feed was placed on steel mesh forming a 75° angled with the bottom of the feeder (Figure 2). For each 10-min test, two different feeders were opened. The combination of feed type (poplar leaves or cut grass) and feeder position (elevated feeder and floor-level feeder), and the side of the feeder were changed for each goat. The step was covered with a mesh (chicken wire) to prevent slipping.

**Figure 2:**
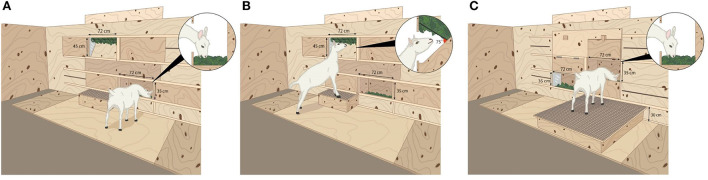
Detailed drawings of the three different feeding settings. **(A)** Floor-level feeder: head of the goat lowered during feeding, ground-oriented, to mimic grazing. **(B)** Elevated feeder: the head and the body of the goat were pointing upwards with two front hooves on a step (25 or 30 cm height) to mimic browsing. The step was covered with a mesh to prevent slipping. **(C)** Platform-level feeder identical to the floor-level feeder, but accessible by putting all four hooves on a platform (30 cm height). Head of the goat was lowered during feeding, ground-oriented, to mimic grazing. The platform was covered with a mesh to prevent slipping. Settings A and B were used in Exp1, while settings A and C were used in Exp2.

The testing arena had solid plywood walls and a concrete floor that was partially covered by plywood in the area directly in front of the feeders (Figure 1). For each test, a goat was led into a corridor (W: 0.35 m, L: 1.4 m, H: 1.8 m), where it waited 30 s between two closed doors, before gaining access to the testing arena. Along the long wall of the testing arena opposite the entry door, two feeder types could be independently opened and closed on each side. Two feed types were used in the tests: freshly cut, mixed grass (GRA) and poplar leaves (POP: *Populus deltoids x Populus nigra*). The leaves were roughly detached from the larger branches, but there stems and small branches remained. GRA was cut once a day using a front mount mower (Disc S Alp, SIP) and a wagon (Bergman 28S). In Exp1, due to equipment availability, feed was delivered between 0830 and 0930 h; in Exp2 all deliveries were at 0930 h. POP was cut manually using a hedge trimmer (Telescopic Pole Hedge Trimmer, 0HT1860S, RYOBI) twice a day and delivered at 0930 and 1300 h. Both feeds were offered in both experiments. In Exp1, feeding posture differed between feeder options. Feed was presented in a floor-level feeder (Figure 2A), and in an elevated-level feeder with a step (W: 0.76 m, L: 0.40 m, Figure 2B) on which the goats could only put their two front hooves. In Exp2, feeding height, but not feeding posture, differed between feeder options. Feed was presented in two of the same floor-level feeders as in Exp1, with one of these feeders raised on a platform (W: 1.2 m, L: 1.2 m, H: 0.30 m; Figure 2C) which the goats could stand on with all four hooves (platform-level feeder). Each experiment consisted of a habituation period (four days in Exp1 and two days in Exp2) followed by two 4-day periods of testing. To avoid side bias, an 8x8 Latin Square design ensured that each feed type and feeder combination was presented on both sides of the test arena (Left or Right) to every goat (Table 1). All tests (including habituation periods) lasted 10 min per goat.

**Table 1:** Example of testing schedule for one goat as determined using an 8x8 Latin Square. The elevated feeder was positioned 1.03 m from the ground and the floor-level feeder was positioned 0.30 m from the ground (see Figure 1).

	**Elevated feeder left-hand side**	**Elevated feeder right-hand side**	**Floor-level feeder left-hand side**	**Floor-level feeder right-hand side**
Day 1	GRA			POP
Day 2		GRA	GRA	
Day 3		POP	GRA	
Day 4	POP			POP
Day 5		GRA	POP	
Day 6	GRA			GRA
Day 7	POP			GRA
Day 8		POP	POP	

GRA, cut grass; POP, poplar leaves. Sequences across the days were determined by pseudo-randomization (in a balanced way) for each goat.

### Experiment 1

#### Feeders

Feeders were made of plywood, with an acrylic plate on one side to allow a camera view inside the feeder. The floor-level feeder was accessible *via* an opening at 0.30 m from the ground, with the feed raised to 10 cm above the ground level, which allowed the goat to pass its head through and to eat with the head facing down in a grazing posture (Figure 2A). The elevated-level feeder had an opening (1.03 m from the ground) which allowed the goat to eat with its head angled upward, and the step was adjusted based on the height of each goat. Using six pilot goats from the main herd to determine optimal step height, two steps were developed. Enrolled goats measuring between 77 and 86 cm at the shoulder were given a 30 cm high step (*n* = 7), while goats measuring between 88 and 92 cm were given a 25 cm step (*n* = 9). Due to some slipping noticed on day 3 of the first testing period, mesh (chicken wire, mesh opening 6 mm) was added to all steps. The bottom of the elevated-level feeder was angled at 45° and the feed was placed on steel mesh, forming a 75° angle with the bottom of the feeder (Figure 2B). Sliding doors controlled by technicians from outside the pen made it possible to close the feeders remotely and independently of each other; this allowed for the presentation of all feeder combinations and prevented the goats from continuing to eat after the 10-min test period.

#### Habituation and testing schedule

On the first day of habituation (day-8), pairs of goats were given arena access with the feeders closed. On day-7 and day-6 of habituation, the same pairs were presented with access to either two floor-level or two elevated-level feeders. Feeder presentation was pseudo-randomized for each pair of goats (each pair of goats met each presentation once, but the order of the presentation was random), so that there was the same number of pairs with each type of presentation. To ensure goats interacted with the feeders, each feeder contained two familiar feeds, which the goats routinely receive in the winter as supplemental feed: 1 kg of alfalfa silage (Equifibre^®^ Lucerne Pro, Dunstan Horse Feeds, Ltd., Hamilton New Zealand) and 160 g of pellets (Fiber Grow, Dunstan Horse Feeds, Ltd., Hamilton New Zealand). Over the following four days (day-5 to -2), goats were habituated alone to the feeders in the same manner. Feeder presentation (two floor-level or two elevated-level feeders) was determined in a balanced way for each goat. On the final day of habituation (day-1), goats were presented with one floor-level and one elevated-level feeder, sides allocated randomly with the same number of goats per side. The goats were considered to be fully habituated when they interacted (i.e., put their heads in the feeder) and/or ate at least once in each feeder. Beginning the following day (considered as day 1) and continuing for the next 7 days, an 8x8 Latin square protocol was followed for each goat, presenting treatments as outlined in Table 1, with the order of the feeding height/posture and feed type combinations differing among goats. Feeders contained either 2 kg of GRA or 1.5 kg of POP; these quantities were defined after pilot testing with five goats from the main herd to ensure both feed types were available *ad libitum* during the 10 min tests.

### Experiment 2

#### Feeders

The same testing arena was used as in Exp1 (Figure 1). The floor-level feeder was the same (Figure 2A), whereas the elevated feeder was replaced by a floor-level feeder on top of a platform (Figure 2C). The opening of the feeder was positioned 0.60 m from the ground. Access to each feeder, as well as opening and closing, was accomplished using sliding doors across the openings.

#### Habituation and testing schedule

Exp2 habituation began immediately after the end of Exp1 as the goats were already familiar with the testing procedures and the floor-level feeder. Goats were individually presented with two platform-level feeders over 2 days of habituation. All other details (goat order, quantity of feed presented and Latin square design) were repeated from Exp1.

### Feed intake and quality

After each goat completed a test, feed orts from inside and outside (step and floor) each feeder were collected separately and weighed to calculate feed intake in fresh matter. In both experiments, each day, feed samples were taken 15 min before goat 1 and goat 9 began testing, and 30 min after goat 8 and goat 16 ended testing, with 1 h of break between goat 8 and goat 9. All samples were frozen immediately at −20°C. To allow for accurate soluble sugar analysis, Exp2 included a duplicate sample frozen in liquid nitrogen. These samples were put in a polystyrene box and covered completely with liquid nitrogen. Samples remained in this closed box for 5 to 10 min, and after the liquid nitrogen evaporated, the samples were placed in the freezer (-20°C). After this, a sublimation process was used to dry the samples.

In order to determine whether pooling of samples would be appropriate, we arbitrarily chose 2 days in the middle of Exp1 (the last day of the first week and the first day of the second week of the experiment) to analyze separately (i.e., four samples per day for leaves because of two daily deliveries and two samples per day for cut grass) by wet chemistry analysis (Nitrogen, Dry Matter, Crude Protein, Acid Detergent Fiber, Neutral Detergent Fiber, Soluble Sugars, Starch; Hill Laboratories, Hamilton, New Zealand). These analyses determined that the chemical components were stable throughout the day and the remaining samples from Exp1 and those from Exp2 of the same feed type were therefore pooled per day before being chemically analyzed. The average chemical compositions per experiment are presented in Table 2.

**Table 2:** Mean (SD) chemical composition of the feed (GRA: cut grass, POP: poplar leaves) used in Experiment 1 and Experiment 2.

	**GRA**	**POP**	**Hay ^a^**	**GRA**	**POP**	**Hay^a^**
	**Exp 1**			**Exp 2**		
Dry matter (DM, %)	28.95 (2.76)	41.15 (1.98)	88.25 (0.39)	28.44 (1.97)	39.76 (2.39)	87.35 (2.51)
Nitrogen (% DM)	2.15 (0.24)	2.21 (0.03)	1.69 (0.51)	2.20 (0.04)	2.37 (0.19)	1.65 (0.14)
Crude protein (% DM)	13.42 (1.39)	13.75 (0.29)	10.61 (3.20)	13.87 (0.30)	14.76 (1.12)	10.36 (0.94)
ADF (% DM)	29.65 (0.78)	23.00 (1.63)	34.24 (0.12)	30.09 (0.88)	18.16 (3.34)	34.51 (1.26)
NDF (% DM)	47.45 (0.60)	33.27 (1.84)	62.04 (0.37)	48.29 (2.18)	27.21 (4.50)	63.08 (1.48)
Soluble sugars (% DM)	6.70 (0.42)	11.59 (0.23)	NA	6.55 (0.29)	12.78 (1.34)	NA
Starch (% DM)	1.70 (0.07)	2.64 (0.09)	NA	0.94 (0.05)	1.47 (1.33)	NA

a Hay provided in the waiting pens adjacent to the testing arena.

### Behavior

The test arena was recorded using an NX Witness Video Management System (Network Optix, Burbank, CA, USA) and three color 4-megapixel cameras with 2.8 mm, f/2 lenses (DS-2CD2342WD-I, Hikvision, Hangzhou, China). One camera was positioned at the side of each feeder, and two positioned at the back and front of the testing arena. Videos were extracted from the system and combined into a single video using Shotcut (Meltytech, LLC) software. There were 16 videos per goat, 252 videos in total (4 videos were lost due to technical issues). Interact (Mangold, Germany) was used to code behaviors and goat location following an ethogram (Table 3). Inter-observer reliability was tested using five videos selected randomly and with Cohen's Kappa Index output from Interact (all behaviors, κ = 0.84 ± 0.07 between the two observers). The remaining videos were coded by a single observer. Intra-observer reliability was assessed using 15 videos, selected randomly, and watched twice (Cohen's Kappa Index as calculated by Interact; all behaviors, κ = 0.79 ± 0.14).

**Table 3:** Ethogram of behaviors coded for every goat during every 10-min test in Experiments 1 and 2.

**Behavior**	**Category**	**Description of activities**
Sniffing	Non-feeding directed	Muzzle is close to ground or wall. Goat may move.
Scratching	Non-feeding directed	Goat uses a foot in repeat movement against a part of the body or wall or the ground, or its mouth against a part of the body.
Exploring elevated or platform-level feeder	Exploration	Goat explores with muzzle in the elevated or platform-level feeder during more than 2 secs, but the goat does not eat and does not have feed in mouth.
Exploring floor-level feeder	Exploration	Goat explores with muzzle in the floor-level feeder during more than 2 secs, but the goat does not eat and does not have feed in the mouth.
Exploring step/platform with muzzle	Non-feeding directed	Goat explores/nibbles/sniffs the step/platform with its mouth/nose.
Rearing	Non-feeding directed	Goat stands with one or both front limbs on wall of arena.
Feeding from elevated or platform-level	Feeding	Head in or out of the elevated or platform-level feeder using lips and teeth to manipulate and obtain feed, including chewing and swallowing. Goat may move.
Feeding from floor-level	Feeding	Head in or out of the floor-level feeder using lips and teeth to manipulate and obtain feed, including chewing and swallowing. Goat may move.
Walking	Non-feeding directed	Goat moves at least two of its legs from a standing position, moving forward or backward.
Eating on the ground	Other	Goat eats feed that has been spilled on the ground or on the step/platform. Goat may move.

### Statistical analysis

#### Data handling

All statistical data handling and analyses were performed with R (version 3.6.1, R Core Team, 2019) and its specific packages [lme4 package: (24), emmeans package (25)], using goat as the experimental unit. For each experiment, each goat had two replicates of the four feeding height/posture and feed type combinations (i.e., the combinations were presented on the left and on the right, resulting in each goat completing eight tests total in each experiment; Table 1). If a goat did not eat at all, the replicate was considered as missing data because no preference could be established; this occurred for 10 goats in total, for 11 of 128 tests in Exp1 (one in POP/POP, 10 in GRA/GRA), and 11 of 128 tests in Exp2 (all in GRA/GRA).

Two data sets were prepared. The first was at the feeder level, with information on the duration of feeding and the quantity of feed ingested by the goats from each feeder, while the second data set was at the goat level, with information on the overall feed intake and the behaviors performed by the goats during the test period.

#### Dataset 1: Feeder level

The time spent feeding and the quantity of feed eaten were highly positively correlated using a Pearson's correlation test, thus analysis was carried out only on the quantity of feed eaten. The quantity of fresh feed consumed is expressed in grams of dry matter (DM). Using a Wilcoxon test, the side of presentation (left or right) did not significantly affect feeder choice. Side of presentation was also balanced in the Latin square design, and side was therefore not considered in the model. Data were analyzed using the following mixed-effect model: feed intake in one feeder as the response variable, goat as the random effect and three fixed effects: feed type (POP vs. GRA), feeder position (feeding posture: elevated-level vs. floor-level for Exp1; feeding height: platform-level vs. floor-level for Exp2) and choice (GRA/GRA and POP/POP vs. GRA/POP and POP/GRA). All interactions, up to the three-way interaction between the three predictors (feed type^*^feeder position^*^choice), were all retained.

#### Dataset 2: Goat level

Four different ways of measuring goat preference from observed behaviors were considered: 1) feed intake (summed from both feeders), 2) number of changes between feeders (i.e., feeding/exploring at one feeder and switching to the other), 3) first feeder visited (i.e., the first feeder the goat approached during each test, regardless of any feed consumed from it), 4) latency to visit first feeder.

Durations for sniffing, exploring step/platform, scratching, walking, and rearing behaviors were summed together as ”duration of non-feeding directed activity“ for each goat (Table 3). The durations of exploring the different feeders were summed together as “duration of exploration behaviors.” The behavior “duration of eating on the ground” was considered separately. The time spent doing each activity was transformed into a proportion of time spent doing each activity out of the total time the animal was monitored (10 min).

A mixed-effect model was used to determine the effect of feed type and feeder position combinations on the outcome variables: feed intake, number of feeder changes, and latency to first visit. The combinations assessed were as follows: GRA/GRA vs. POP/POP vs. GRA/POP vs. POP/GRA (where the numerator indicates elevated-level (Exp1) or platform-level (Exp2) feeder and the denominator indicates floor-level feeder). The proportion of the test spent performing exploration behaviors, non-feeding directed activity, and eating on the ground were considered fixed effects, and goat was fit as a random effect. Side was not considered in the model. For the outcome variable of first feeder visited, Chi-square tests were performed, using the null hypothesis that there were no differences in the first feeder visited (elevated level vs. floor-level for Exp1; platform-level vs. floor-level for Exp2) for the different combinations of feed type and feeder position.

For both datasets, distributions of model residuals were checked for normality and homoscedasticity. A log_10_ transformation was performed on the latency to approach the first feeder to meet these requirements. The F-test was used to evaluate the statistical significance of the fixed effects in the models. Significant interactions were tested *post-hoc* using pair-wise Bonferroni corrected comparisons. Results are presented as least squares means with standard errors unless otherwise stated. Significance threshold used was *P* < 0.05.

## Results

### Feeder level

Goats consumed considerably more poplar leaves compared with cut grass in both Exp1 (POP: 188 ± 6.52 g of DM, GRA: 20.3 ± 7.19 g of DM, F_1, 226_ = 299, *P* < 0.001) and Exp2 (POP: 191 ± 6.15 g of DM, GRA: 0.231 ± 6.91 g of DM; F_1, 219_ = 451, *P* < 0.001).

**Figure 3:**
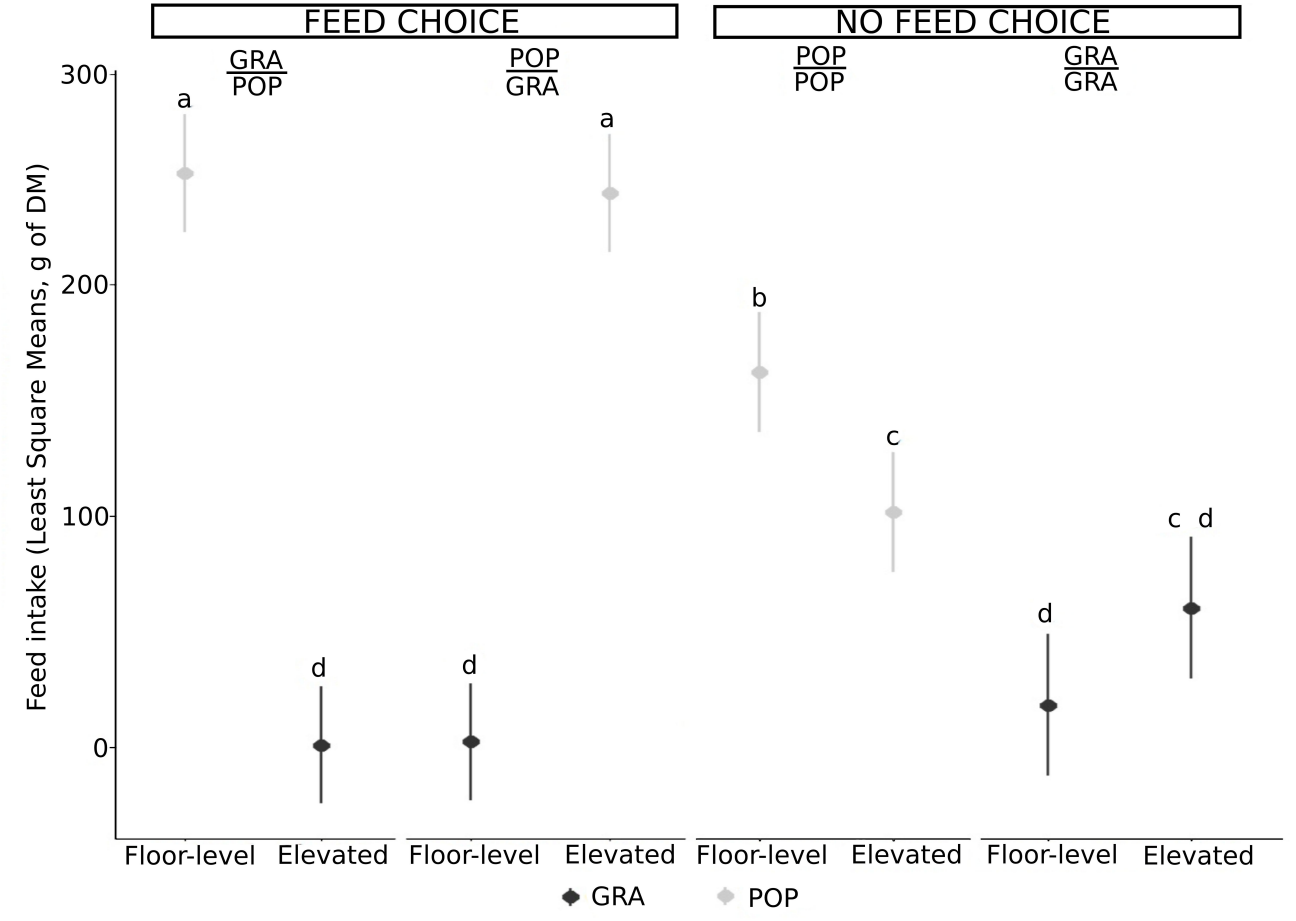
Least Square (± SE) of the quantity of each feed type (GRA: cut grass and POP: poplar leaves) eaten from each feeder (elevated feeder and floor-level feeder) per goat (*n* = 16, with two repetitions per individual) for Exp1, depending on the combination of feed type and feeding posture (Elevated/Floor: POP/POP, GRA/GRA, POP/GRA and GRA/POP), grouped according to the possibility of choosing a feed item or not. Tests where no feed was eaten have been excluded (*n* = 2 GRA/GRA). The dot and bars represent the LSmean and standard error of each combination. Significant differences indicated by the model (*P* < 0.05) are represented by different letters. The interaction between the feed type, the feeding posture, and the possibility to choose between feed or not was significant.

In Exp1, when the goats had the choice of eating either poplar leaves or grass, they showed a preference for the POP regardless of whether presented in the floor-level feeder (GRA/POP; df = 211, t.ratio = 13.5, *P* < 0.001) or presented in the elevated-level feeder (POP/GRA; df = 211, t.ratio = 13.0, *P* < 0.001; Figure 3). More precisely, all goats ate almost exclusively from the low-level feeder containing leaves when grass was offered in the elevated feeder. When the elevated feeder contained the leaves, all but one goat ate from this feeder almost exclusively, **Figure 5**). No matter the feeding posture (floor-level vs. elevated-level feeder), the quantities of leaves consumed were similar in these two combinations (df = 211, t.ratio = 0.478, *P* = 1.0; Figure 3). Goats ate minimal grass from both feeders (df = 211, t.ratio = 0.069, *P* = 1.0; Figure 3). In contrast, when only poplar leaves were offered (no feed choice), goats consumed more from the floor-level feeder than from the elevated level feeder (df = 211, t.ratio = 3.24, *P* = 0.039; Figure 3), while when only cut grass was available (no feed choice), goats did not show any preference between the two feeding postures (df = 211, t.ratio = 1.91, *P* = 1.0; Figure 3).

**Figure 4:**
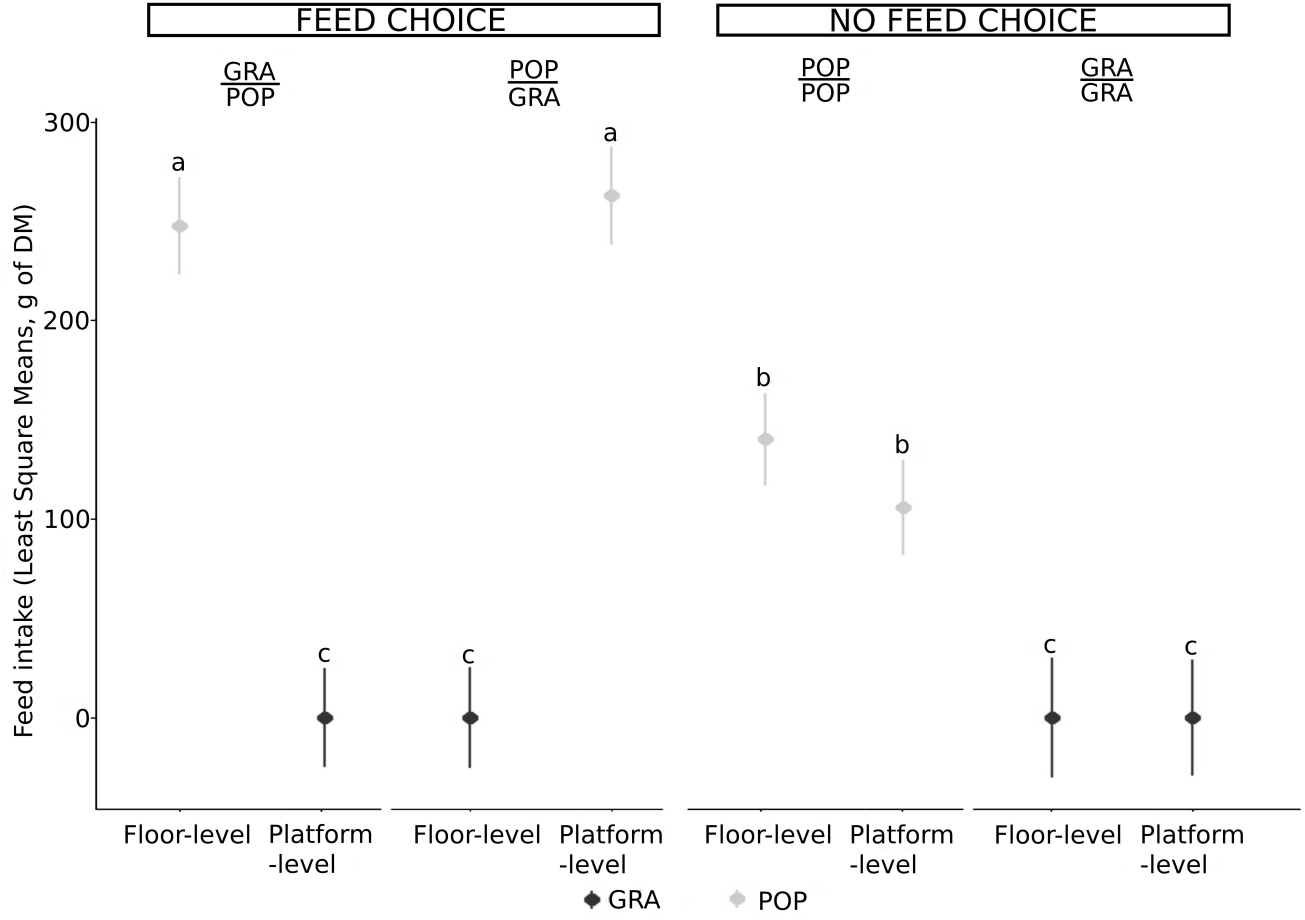
Least Square means (± SE) of the quantity of each feed type (GRA: cut grass and POP: poplar leaves) eaten from each feeder (platform-level feeder and floor-level feeder) per goat (*n* = 16, with two repetitions per individual) for Exp2, depending on the combination of feed type and feeding height (Platform/Floor: POP/POP, GRA/GRA, POP/GRA and GRA/POP), grouped according to the possibility of choosing a feed item or not. Tests where no feed was eaten have been excluded (*n* = 4 GRA/GRA). The dot and bars represent the LSmean and standard error of each combination. Significant differences indicated by the model (*P* < 0.05) are represented by different letters. The interaction between the feed type and the possibility to choose between feed or not was significant.

**Figure 5:**
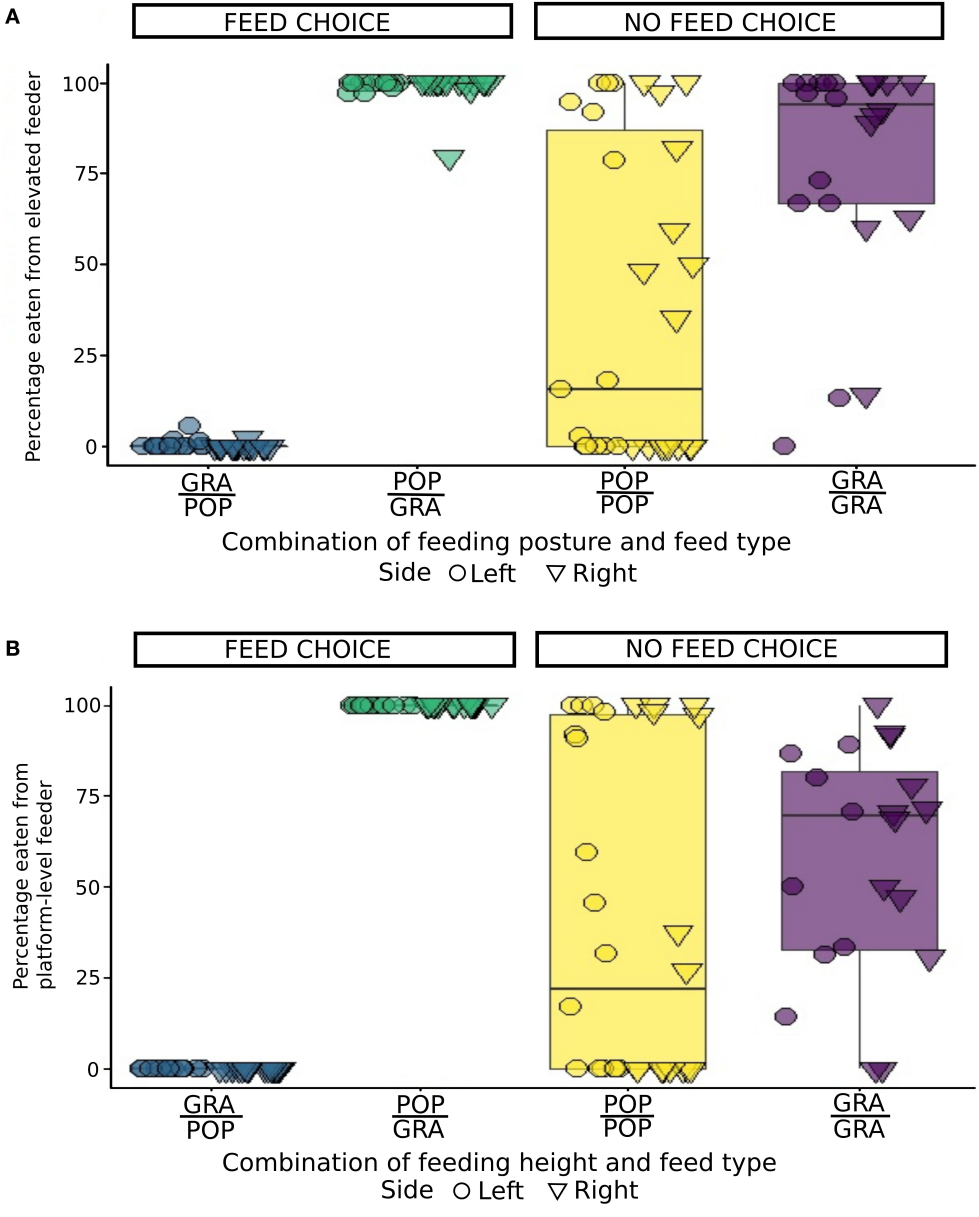
Quantity of feed eaten from the highest of two feeders as a percentage of the total amount of feed eaten per goat (*n* = 16, with two repetitions per individual) for Exp1 **(A)** and Exp2 **(B)**, depending on the combination of feed type and feeding posture (Elevated/Floor: POP/POP, GRA/GRA, POP/GRA and GRA/POP) or feeding height (Platform/Floor: POP/POP, GRA/GRA, POP/GRA and GRA/POP). Tests where no feed was eaten have been excluded (Exp1: *n* = 2 GRA/GRA; Exp2: *n* = 4 GRA/GRA). The dot and bars represent the mean and standard deviation of each combination presented on each side (right or left).

In Exp2, goats did not show a preference for feeding height (platform- vs. floor-level feeders). Goats ate more leaves than grass in total, whether there was a feed choice (df = 211, t.ratio = 21.2, *P* < 0.001) or not (df = 224, t.ratio = 9.48, *P* < 0.001; Figure 4) and there was no variability between individuals (Figure 5).

Whether in Exp1 or Exp2, when there was no feed choice (GRA/GRA and POP/POP), the variability among individuals in feeder choice was substantial (Figure 5). For instance, when only leaves were available, while the overall mean percentage eaten from the elevated level feeder was low (e.g., <25%), there were clearly some individuals that preferred this feeder.

### Goat level

#### Feed intake

The overall intake depended on the combination of feed type (POP vs GRA) and feeding posture (Exp1: floor-level vs elevated-level feeder; F_3, 103_ = 13.0, *P* < 0.001) or feeding height (Exp2: floor-level vs platform-level feeder; F_3, 100_ = 65.7, *P* < 0.001), on the duration of non-feeding behaviors (Exp1: F_1, 105_ = 68.8, *P* < 0.001; Exp2: F_1, 101_ = 11.6, *P* < 0.001), and in Exp1 on the duration of explorative behaviors (F_1, 104_ = 4.24, *P* = 0.042). Goats consumed less overall in GRA/GRA than with the other combinations (Table 4). In addition, the more time the goats spent exploring (in Exp1) or performing non-feeding activities (in both Exp), the less they ate (Exploring: Exp1: t = −7.14, df = 123, *P* < 0.001, r = −0.541; non-feeding activities: Exp1: t = −10.8, df = 123, *P* < 0.001, r = −0.698; Exp2: t = −9.97, df = 120, *P* < 0.001, r = −0.673).

**Table 4:** Least Square means (± SE) of total and feed type intakes, feeder changes, and latency to visit the first feeder during the 10-min tests, for 16 goats depending on the combination of feed type and feeder position in Experiments 1 and 2, respectively.

	**Exp 1**				**F and P values**	**Exp 2**				**F and P values**
	**POP** **POP**	**GRA** **GRA**	**POP** **GRA**	**GRA** **POP**		**POP** **POP**	**GRA** **GRA**	**POP** **GRA**	**GRA** **POP**	
Total feed intake (g of DM)	*220 ± 19.5 **a***	*119 ± 21.7 **b***	*206 ± 19.9 **a***	*218 ± 19.2 **a***	F = 13.0 *P* < 0.001	*234 ± 18.3 **a***	*28 ± 20.4 **b***	*235 ± 18.3 **a***	*223 ± 18.3 **a***	F = 65.7 *P* < 0.001
Feeder changes (no.)	*0.500 ± 0.194 **b***	*2.23 ± 0.284 **a***	*0.488 ± 0.188 **b***	*0.365 ± 0.180 **b***	F = 9.19 *P* < 0.001	*0.677 ± 0.198 **b***	*1.83 ± 0.307 **a***	*0.353 ± 0.202 **b***	*0.308 ± 0.199 **b***	F = 5.16 *P* = 0.002
Back transformed latency means (s)	*8.41 ± 1.23 **a***	*8.09 ± 1.27 **a***	*9.44 ± 1.23 **a***	*10.3 ± 1.22 **a***	F = 1.17 *P* = 0.326	*7.85 ± 1.22 **a***	*12.5 ± 1.28 **a***	*9.46 ± 1.22 **a***	*7.08 ± 1.22 **a***	F = 2.42 *P* = 0.070

For each variable within each experiment, significant differences (P < 0.05) between means are represented by different letters. Statistics refer to significant effects of combination of feed type and feeder position. POP, poplar leaves; GRA, cut grass.

#### Changes between feeders

In both experiments, the combination of feed type and feeding posture or feeding height (Exp1: F_3, 108_ = 9.19, *P* < 0.001; Exp2: F_3, 115_ = 5.16, *P* = 0.002), the duration of exploratory behaviors (Exp1: F_1, 118_ = 18.5, *P* < 0.001; Exp2: F_1, 115_ = 10.3, *P* = 0.002) and the duration of non-feeding activity in Exp1 (F_1, 117_ = 6.57, *P* = 0.012) were associated with the number of changes between feeders. Goats made more feeder changes in GRA/GRA compared to the other combinations (Table 4). In addition, goats made more feeder changes as the duration of non-feeding activities (Exp1 only: t = 4.28, df = 123, *P* < 0.001, r = 0.360) and explorative behaviors (Exp1: t = 9.35, df = 123, *P* < 0.001, r = 0.645; Exp2: t = 6.70, df = 120, *P* < 0.001, r = 0.521) increased.

#### First feeder visited

**Figure 6:**
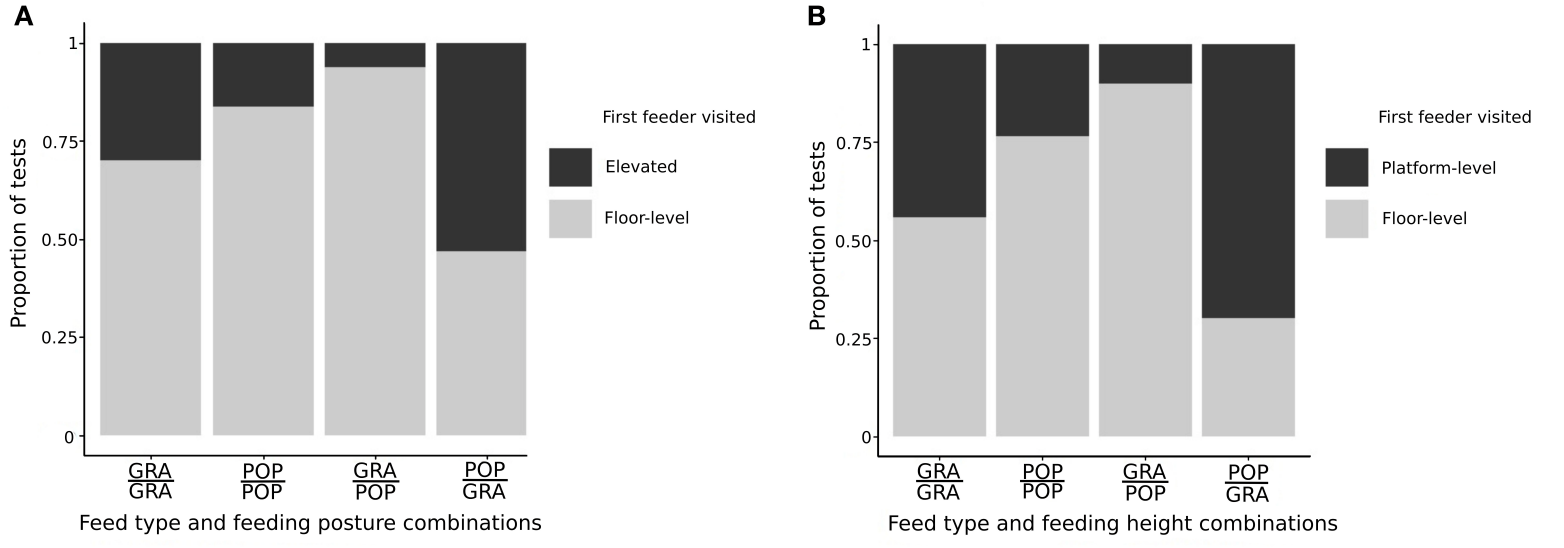
First feeder visited expressed as a proportion of tests where visits were made (*n* = 149), for each combination of feed type and feeding posture or height, for **(A)** Exp 1 (elevated feeder and floor-level feeder) and **(B)** Exp2 (platform-level feeder and floor-level feeder).

The combination of feed type and feeding posture or feeding height was also a good predictor of the first feeder approached by the goat (Figure 6). Goats visited the floor-level feeder first for nearly all combinations presented (Exp 1: POP/POP: X^2^ = 14. 2, df = 1, *P* < 0.001; GRA/GRA: X^2^ = 4.80, df = 1, *P* = 0.029; GRA/POP: X^2^ = 24.5, df = 1, *P* < 0.001; Exp 2: POP/POP: X^2^ = 9.32, df = 1, *P* = 0.002; GRA/POP: X^2^ = 21. 1, df = 1, *P* < 0.001), except for the POP/GRA combination in Exp2 where they first visited the platform-level feeder (X^2^ = 4.17, df = 1, *P* = 0.041). They showed no preference for the first feeder visited with the POP/GRA combination in Exp1 (X^2^ = 0.125, df = 1, *P* = 0.724) and GRA/GRA in Exp2 (X^2^ = 0.533, df = 1, *P* = 0.465).

#### Latency to visit first feeder

The latency to approach the first feeder was not affected by any of the combinations or activities recorded in either Exp1 or Exp2 (Table 4).

## Discussion

Goats are known to prefer elevation when resting (19) and are often seen browsing on foliage (i.e., eating shrubs, trees, and other herbaceous matter, at or above eye-level). The present study aimed to investigate preference for feeding posture (‘grazing' at floor level vs. “browsing” above eye level) and feeding height (grazing posture at either floor level or raised off the ground). Moreover, it attempted to determine if the preference for the feeding posture or feeding height would be dependent upon feed type (grass which would be typical of a grazing situation vs. leaves which would be more typical of a browsing situation).

Interestingly, we found that goats consumed more poplar leaves compared to cut grass, and that this preference was not affected by the feeding posture or if the feeder was raised off the ground. This preference for leaves over grass is likely to be associated with the former containing low fiber (NDF and ADF), high soluble sugars and high starch; these factors translate into better digestibility, a better source of energy, and result in less gut fill in the rumen compared to grass (26–29). Hunger was likely not a significant contributor to the decisions made by the goats during testing, since goats had free access to hay prior to testing and latencies to approach the first feeder were similar for the different combinations of feed type and feeder position. Therefore, the need to meet nutritional requirements is unlikely to account fully for this preference for leaves. This assumption was supported by the finding that in several cases goats did not eat at all and consumed overall less when only cut grass was offered. The preference for poplar leaves may therefore be due to a difference in palatability between the two diets (30). For instance, satiated sheep ingested a substantial meal when a newly offered forage was sufficiently palatable (31); this behavior depended on the forage itself, as well as on what the animals were eating previously and their state of hunger. It can therefore be assumed that poplar leaves, a feed not typically available to goats used in this study, were more palatable than cut grass.

When entering the testing pen, goats most often visited the feeder containing leaves first. Goats are likely to use visual and olfactory cues to detect subtle differences between feeds and make feed choices (32, 33). Indeed, goats, like sheep, are capable of distinguishing colors and shades of gray (34), with a visual field about 270° and a binocular field being about 45° (35). Laboratory experiments have shown that goats possess numerous cognitive capacities (e.g., foraging, navigational or social cognitive capacities, (36), including the ability to categorize feed items and recall preferred feed types and locations. When approaching the feeders, the goats could see and smell the content of each feeder, and likely could discriminate between grass and leaves before reaching the feeder. In view of the goats' preference for leaves, it was therefore not surprising that the feeder containing leaves was visited first whenever there was a choice, except when leaves were in the elevated feeder (POP/GRA) in Exp1, where no first visit preference was evident. Any choice expressed is likely to reflect a trade-off between palatability and accessibility of the feed. For instance, Ginane et al. (37) gave heifers the choice of either ingesting poor quality forage provided *ad libitum* directly at the entrance or having to walk through the testing area to be rewarded with good quality, but limited quantity of forage. The authors found that the heifers crossed the arena to consume the good quality forage, but once it was consumed, the heifers preferred to consume the lower quality (but more accessible) forage rather than returning to the entrance to consume the higher quality forage. A similar method could be used to measure the effort that goats are willing to make to obtain the most desired resource.

A preference for feeding posture was investigated when both feeders contained the same feed. Indeed, Neave et al. (18) reported a strong preference for feeding from an elevated feeder, with goats consuming more from, and actively competing for access to, a feeder identical in design to our Exp1 elevated feeder. In Exp1 we did not observe a preference for the elevated feeder, but for the floor-level feeder. A number of factors could have contributed to this finding. First, we determined preference from a short test; upon entering the testing pen, the goats had 10 min to approach the feeders and consume what they could in that time frame. Therefore, it is unsurprising that goats consumed quite a bit of their preferred feed (poplar leaves, regardless of feeder type). They also visited the floor-level feeder first in Exp1; this may relate to the floor-level feeder being closest to eye level, so when entering the pen, the goats could clearly see this opening without looking up. Upon seeing the poplar leaves in the floor-level feeder, the goat would start eating, consuming most of the total intake from this trough, with little incentive to change to the higher feeder. We acknowledge that this line of sight directly from the entrance to the floor-level feeder was a limitation of our study set up, and we would suggest future work in this area plan to visually obscure the feeders to ensure goats had to approach them to see their contents. Finally, we acknowledge that due to the need to contain feed, a mesh grid was used in this elevated feeder (that goats had to pull the feed through). Although this was the same design as Neave et al. (18), the feed used in our study was less uniform than that of Neave. So, it is possible that accessibility contributed to the preference for the floor-level feeder.

It has been suggested that feeding at high levels may lead to higher energy costs due to the necessary posture, as well a greater need for vigilance due to the vulnerability of the goat in the bipedal position (38). In addition, the shape of the elevated feeder required the goats to pull the feed through the mesh to retrieve it. Nonetheless, it is unlikely that goats calculate energy efficiency when faced with a choice of floor- vs. elevated-level or platform-level feeders. Indeed, in our study it was likely the length of test that influenced where and how much was consumed; other studies [e.g., (18, 20)] observed the goats for at least 24 h. A second point of difference between the present study and others which found a preference for height is that our experiments were conducted on goats tested individually. Aschwanden et al. (20) showed that providing a floor-level feeder and a platform-level feeder allowed goats to increase the distance between individuals and thus reduce agonistic behaviors during feeding. Conversely, provision of a feed at different heights actually increased the level of competition for the highest feeder when Neave et al. (18) observed goats in groups of three. Regardless, it is likely that if our goats had been in a group, we would have seen a greater use of the elevated- or platform-level feeder. Finally, while (39) used a highly competitive situation, their study nonetheless showed that different personalities played a role in the use of elevated feeders; thus, we suggest that some of the individual variation (e.g., some goats not consuming any feed in the testing pen) could be explained by considering personality traits.

In contrast to that seen when offered leaves, when only grass was offered, similar (and small) amounts of grass were consumed from both feeder options (in Exp1 and Exp2) and goats approached the platform-level feeder first (in Exp 2). The latter might be explained with the same theory as for why the goats approached the floor-level feeder when presented with only leaves; goats likely saw that grass was presented in this feeder as they entered and therefore opted to jump onto the platform (which may have obscured the direct line of sight into the platform-level feeder) immediately to investigate what was offered in that feeder. Furthermore, there were more changes between the two feeders when they both contained grass than in the other combinations. This exploration of both feeders could reflect an attempt to find either another preferred feed source, or another feeder position. The search for another feed was supported by the finding that some goats did not consume feed at all; of all 22 instances of non-consumption, all but one was when only grass was presented. In nature, goats rely on social information to determine patch quality, but when foraging alone, they must rely on direct sampling of food patches to identify differences in quality (40). Previous experimental work in goats has also shown goats will change between food sources when faced with low quality feed; when goats were presented with low- or high- quality feed in the arms of a T maze, they would often switch to the other arm if they initially chose the low-quality arm (41). Similarly, in cattle, Huzzey et al. (42) reported that heifers fed a diet with lower energy density compared to that of a previous period increased their changes between feeding stations. The authors indicated that these behavioral changes may reflect the expectation of better-quality feed at another feeding station. Conversely, when the feed had a higher energy density than previously received, the number of feeding station changes was reduced, suggesting that the heifers were content with maintaining consumption of the better feed. The balanced design of our experiment meant that the majority of goats would have experienced leaves in one or both of the feed troughs prior to being presented with the GRA/GRA combination, which could have led them to expect finding a better-quality feed in the other feeder. In addition to these results, visually, we noticed that there was a large inter-individual variability in feeding posture and height preferences; while it is possible that some of the variability was based on previous experience (i.e. what the goat received in the previous day's test), we suggest individual goat personality could also be a contributing component to this variation. Indeed, it has been shown that different goat personality types have an impact on feeding behavior and especially on feeding duration (39); in some cases, these differences were linked to how the feed was presented. Goats are classified as both browsers and grazers and can be seen eating in different postures (8, 12, 19, 43, 44). Although different factors can influence the feeding posture (e.g., a bipedal stance would not be used without browse present), we suggest that this is driven by individual differences in behavior. Large inter-individual variability underlines the importance of providing multiple options in terms of feeding posture and height, and in feed type, to allow goats to make choices.

In the present study, the ability to choose among two types of feed did not lead to increased feed intakes overall. This is likely a combination of the poplar leaves being greatly preferred, and because the test was of too short a duration. Ginane et al. (45) found an increase in intake in a choice situation compared to a no-choice situation in heifers offered different types of hay. Their result indicates that motivation to eat is reinforced by the diversity of feeds offered. Allowing animals to choose between different feeds provides them with agency. The potential to stimulate intake by feed diversity, combined with the preference for leaves, as well as goats' natural ability to browse, may have animal welfare implications for current feeding systems; for instance, in New Zealand most feeding regimes consist of freshly mowed, ”cut and carried“ grass (22), while in Canada homogenous pelleted feeds or Total Mixed Rations have been reported (21). Indeed, even if an abundant, and on average nutritious, diet is given to the herd, it is not necessarily sufficient to foster the welfare of all the individuals. For example, although nutritional requirements are fulfilled, if cattle housed indoor are not provided with sufficient roughage, oral stereotypies such as tongue rolling may occur (46). Tongue rolling is similar to the natural oral manipulation of grass on pasture and has been suggested to be a response to grazing behavior being thwarted (47). Goats that are housed indoors may face similar thwarted situations and redirect foraging behavior toward their environment; Zobel and Nawroth (36) discuss farmers anecdotally reporting problematic behaviors (e.g., chewing on gate latches, constantly trying to access out-of-reach objects). We suggest that alterations to the environment for housed goats could be both beneficial and feasible, particularly with regard to feeding management. Providing feed raised off the ground has been reported for commercial settings [e.g., (19)]. Providing dietary flexibility, whether in terms of what goats can eat, or how they eat could improve their affective state by allowing them to express preferences or even by increasing the opportunity to use their cognitive capabilities when exploring the environment. A recently suggested option to increase this flexibility would be the provision of automated feeding units; Zobel and Nawroth (36) and (48) for example described task-based access as a means of promoting natural foraging behavior, and to provide a form of cognitive enrichment.

## Conclusion

In conclusion, our study showed that the type of feed affected the preference for feeding position. When given a choice between two feeds, the goats preferred to eat leaves (vs grass), regardless of the feeder position. When only leaves were available, goats ate from the floor-level feeder rather than the elevated-level feeder, however when only cut grass was available, there was no feeding posture preference and goats explored and changed feeders more often, presumably searching for another feed option. We suggest these findings reflect differences in palatability of the leaves and grass. We also caution that our study determined preference by using a short duration test, which likely resulted in their focus being on the feed, and not on the feeding position; a longer testing period is needed to better understand height and posture preference. Most commercial systems feed homogenous, and often grass-based, feeds; we suggest that the goats' strong preference for a feed such as leaves highlights that more effort should be placed in promoting natural feeding behavior, and this may contribute to better goat welfare.

## Data availability statement

The raw data supporting the conclusions of this article will be made available by the authors, without undue reservation.

## Ethics statement

The animal study was reviewed and approved by Ruakura Animal Ethics Committee (Hamilton, New Zealand: no. 14680) under the New Zealand Animal Welfare Act 1999.

## Author contributions

MC: made substantial contributions to data acquisition, analysis, interpretation, drafted, revised the work, and wrote the final version. BN and CD-P: made substantial contributions to data analysis and interpretation and revised the work. HF: made substantial contributions to conception of the work and data acquisition and revised the work. RH: made substantial contributions to data analysis and interpretation. BM and KC: made substantial contributions to conception of the work. EO'C: made substantial contributions to data collection. HN: made substantial contribution to the interpretation and revision of the work. GZ: made substantial contribution to conception of the work, data analysis, and interpretation and revised the work. All authors contributed to the article and approved the submitted version.

## Funding

The work was funded by AgResearch Strategic Science Investment Fund (contract C10X1702 with the Ministry of Business Innovation and Employment). MC was supported by the doctoral school ABIES through joint finances from Ministère de l'enseignement supérieur, de la recherche et de l'innovation and INRAE (Institut national de recherche pour l'agriculture, l'alimentation et l'environnement), the latter acting as her employer.

## Conflict of interest

Authors HF, RH, BM, EO'C, KC, and GZ were employed by AgResearch Ltd. The remaining authors declare that the research was conducted in the absence of any commercial or financial relationships that could be construed as a potential conflict of interest.

## Publisher's note

All claims expressed in this article are solely those of the authors and do not necessarily represent those of their affiliated organizations, or those of the publisher, the editors and the reviewers. Any product that may be evaluated in this article, or claim that may be made by its manufacturer, is not guaranteed or endorsed by the publisher.

## Abbreviations

DM: dry matter
GRA: mixed cut grass
GRA/GRA: poplar leaves in both feeders
GRA/POP: mixed cut grass in the higher feeder and poplar leaves in the lowest feeder
POP: poplar leaves
POP/POP: poplar leaves in both feeders
POP/GRA: poplar leaves in the higher feeder and mixed cut grass in the lowest feeder.
